# Continued rise in the use of mid-level providers in US emergency departments, 1993–2009

**DOI:** 10.1186/1865-1380-5-21

**Published:** 2012-05-23

**Authors:** David F M Brown, Ashley F Sullivan, Janice A Espinola, Carlos A Camargo

**Affiliations:** 1 Department of Emergency Medicine, Massachusetts General Hospital, Harvard Medical School, Boston, MA, 02114, USA

**Keywords:** emergency department, workforce, mid-level providers, physician assistants, nurse practitioners, trends, NHAMCS

## Abstract

**Background:**

Emergency department (ED) visits in the US have risen dramatically over the past 2 decades. In order to meet the growing demand, mid-level providers (MLPs) – both physician assistants (PAs) and nurse practitioners (NPs) – were introduced into emergency care. Our objective was to test the hypothesis that MLP usage in US EDs continues to rise.

**Findings:**

We analyzed ED data from the National Hospital Ambulatory Medical Care Survey to identify trends in ED visits seen by MLPs. We also compared MLP-only visits (defined as visits where the patient was seen by a MLP without being seen by a physician) with those seen by physicians only. During 1993 to 2009, 8.4% (95%CI, 7.6–9.2%) of all US ED visits were seen by MLPs. These summary data include marked changes in MLP utilization: PA visits rose from 2.9% to 9.9%, while NP visits rose from 1.1% to 4.7% (both P_trend_ < 0.001). Together, MLP visits accounted for almost 15% of 2009 ED visits and 40% of these were seen without involvement of a physician. Compared to physician only visits, those seen by MLPs only were less likely to arrive by ambulance (16% vs 6%) and be admitted (14% vs 3%).

**Conclusions:**

Mid-level provider use is rising in US EDs. By 2009, approximately one in seven visits involved MLPs, with PAs managing twice as many visits as NPs. Although patients seen by MLPs only are generally of lower acuity, these nationally representative data confirm that MLP care extends beyond minor presentations.

## Findings

In recent years, emergency department (ED) visits have increased dramatically [1], and there are insufficient emergency physicians to care for all of these visits [2]. The physician shortage is likely to continue for several decades [3], particularly in rural areas of the country [4-6]. In order to meet the growing demand, mid-level providers (MLPs) – both physician assistants (PAs) and nurse practitioners (NPs) – were introduced into emergency care. Working alongside emergency physicians, MLPs can improve the efficiency of physicians seeing patients [7]. MLPs are less expensive (both to train and to compensate), and they are capable of managing lower acuity visits. However, at least one report found that MLPs, when working autonomously in EDs, are less likely to follow evidence-based clinical guidelines [8].

An early report on MLP utilization in US EDs examined 1992 data [9]. More recent work evaluated trends from 1993 to 2005 and indicated a growing role for MLPs, both in co-management with physicians and in autonomous patient care [10]. An understanding of more recent trends will inform ED workforce analyses, particularly in the context of the physician shortage. It also will help ED directors to better manage MLP roles as part of ED staffing. In this article, we evaluate MLP participation in US ED visits from 1993 to 2009. We hypothesized that the rising trend noted from 1993–2005 would continue through the end of 2009. Additionally, we hypothesized that the role of PAs would continue to outpace that of NPs as ED providers.

## Methods

We combined data from the ED component of the 1993–2009 National Hospital and Ambulatory Medical Care Survey (NHAMCS) to generate national estimates of ED visits seen by PAs and NPs. Methodological details of the NHAMCS survey are described elsewhere [11]. Briefly, NHAMCS is a four-stage probability sample of visits to US non-institutional general and short stay hospitals, conducted annually by the National Center for Health Statistics. Our institutional review board waived review.

We used the “Providers Seen” fields to analyze all visits seen by PAs and NPs, both with and without documented evidence of physician involvement in clinical care. PA data were collected from 1993 onward, while NP data were from 1995 onward. Physicians were defined as attending, staff, on call, consulting or other physician; on call fellow; or resident/intern. We excluded visits without documentation of MLP or physician involvement.

We performed all analyses using Stata 11.2 (StataCorp, College Station, TX) and utilized an ultimate cluster model to estimate variance. We accounted for NHAMCS variable changes spanning the study period, and all analyses used appropriate survey commands in Stata to account for the sampling frame. Publically available data files and detailed instructions on analysis of the data are available on the NHAMCS website [12].

We compared weighted proportions of characteristics with 95% confidence intervals (CIs) and weighted medians with interquartile ranges (IQR) for visits seen by different provider types. We analyzed annual trends using weighted linear regression with results reported as slopes, which represent the mean change per year in the proportion of the reported characteristic. Trend analyses that included visits seen by NPs were limited to years 1995–2009. A two-tailed *P*-value <0.05 was considered statistically significant.

## Results

From 1993 to 2009, there were approximately 1.8 billion ED visits in the US. During this period, 6.3% (95%CI 5.5–7.0%) of all visits were seen by PAs and 2.5% (95%CI 2.1–2.8%) by NPs. Limiting the analyses to visits without evidence of physician involvement, autonomous care was provided by PAs for 2.6% (95%CI 2.1–3.1%) of all ED visits and by NPs for 1.1% (95%CI 0.9–1.3%).

Table 1 shows ED visit characteristics stratified by type of provider seen. Comparing visits seen by MLPs only to those seen by physicians only, there was variation among several patient, visit, and hospital characteristics. By contrast, characteristics of MLP visits with direct physician involvement were similar to those seen by physicians alone (data not shown).

**Table 1:** Characteristics of US emergency department visits seen by mid-level providers without physician involvement, compared to those seen by physicians only; 1993–2009

	**MLP only**		**Physician only**	
	**No. of visits**	**% (95%CI)**	**No. of visits**	**% (95%CI)**
	*N* = 17,316		*N* = 453,348	
**Patient characteristics**				
Age, years; median (IQR)	17,316	29 (16–44)	453,348	33 (17–52)
Female sex	9,136	53% (52–55)	240,899	53% (53–54)
Race				
White	13,180	76% (72–80)	330,486	75% (74–77)
Black	3,612	22% (18–25)	104,590	22% (20–23)
Other	524	2.6% (2.1–3.1)	18,272	3.1% (2.7–3.5)
Ethnicity				
Non-Hispanic	12,348	72% (67–77)	320,698	71% (69–72)
Hispanic	1,958	10% (8.3–12)	52,416	10% (9.1–11)
Missing	3,010	18% (14–22)	80,234	19% (18–21)
Insurance				
Private	5,980	36% (34–38)	153,281	35% (34–36)
Public	5,731	33% (31–35)	168,147	36% (35–37)
Self-pay	3,225	18% (17–20)	69,486	16% (15–16)
Other/unknown	2,380	13% (11–15)	62,434	13% (13–14)
**Visit characteristics**				
Arrival time^*^				
8 a.m. – 3:59 p.m.	8,029	48% (46–50)	168,175	42% (41–42)
4 p.m. – 11:59 p.m.	7,189	44% (43–46)	168,011	42% (42–43)
12 a.m. – 7:59 a.m.	1,137	7.2% (6.3–8.2)	61,502	15% (15–16)
Arrival by ambulance^+^	854	6.1% (5.1–7.1)	47,960	16% (15–17)
Urgency				
Urgent/Emergent	5,416	34% (30–38)	228,218	60% (58–61)
Nonurgent	9,579	66% (62–70)	153,078	40% (39–42)
Any imaging or X-ray	5,525	33% (31–35)	190,794	43% (42–43)
Any medication ordered	13,387	78% (76–79)	345,734	77% (76–77)
ED length of stay, hours; median (IQR)^#^	12,647	1.9 (1.2–3.0)	256,427	2.4 (1.4–4.0)
Disposition				
Admitted	545	3.1% (2.1–4.2)	65,627	14% (13–14)
Discharged/referred	16,638	96% (95–97)	384,245	85% (85–86)
**Hospital characteristics**				
US region				
Northeast	5,426	25% (16–34)	112,217	19% (17–22)
Midwest	3,524	20% (13–27)	101,524	25% (22–28)
South	5,050	36% (28–44)	153,170	38% (34–42)
West	3,316	19% (13–25)	86,437	18% (15–21)
Urban status				
Urban	13,934	78% (68–87)	387,950	80% (75–85)
Nonurban	3,382	22% (13–32)	65,398	20% (15–25)
Hospital ownership				
Non-profit	13,427	80% (74–87)	318,983	71% (68–74)
Government (non-federal)	2,869	13% (6.9–20)	77,604	15% (13–17)
Private/for profit	1,020	6.6% (4.0–9.1)	56,761	14% (12–16)
	n = 17,316		n = 453,348	

ED, emergency department; MLP, mid-level provider; CI: confidence interval; IQR, interquartile range.

^*^Available 1995–2009

^+^Available 1997–2000, 2003–2009

^#^Available 2001–2009

**Figure 1:**
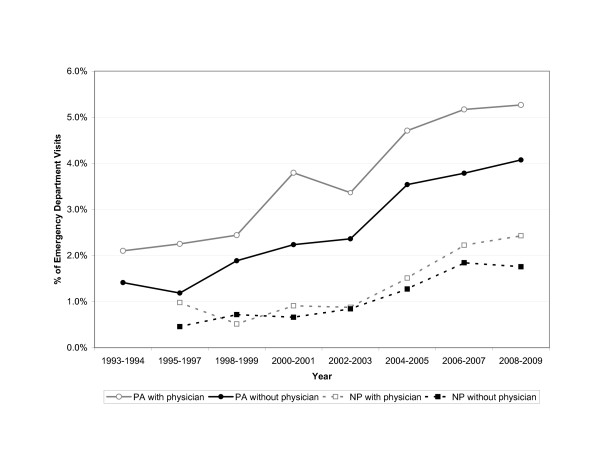
**Trends in emergency department visits seen by mid-level providers with or without direct physician involvement, 1993–2009.** Physician assistant visits without physician involvement (solid circle): slope = 0.002 (95%CI, 0.001 to 0.003). Nurse practitioner visits without physician involvement (solid square): slope = 0.001 (95%CI, 0.0007 to 0.002). Physician assistant visits with physician involvement (empty circle): slope = 0.002 (95%CI, 0.0016 to 0.003). Nurse practitioner visits with physician involvement (empty square): slope = 0.001 (95%CI, 0.0009 to 0.002).

Figure 1 shows a continued rise in usage of both PAs and NPs over the study period. Visits seen by MLPs only increased from 1.2% (95%CI 0.6–1.9%) in 1995 to 5.7% (95%CI 4.1–7.4%) in 2009. Visits seen by MLPs with direct physician involvement increased from 2.8% (95%CI 1.7–3.9%) in 1995 to 8.8% (95%CI 6.7–10.8%) in 2009.

Among visits seen by MLPs only, the proportion of nonurgent visits may have increased very slightly from 70% (95%CI, 56–83%) in 1995 to 71% (95%CI 65–78%) in 2009 (slope, 0.01; 95%CI 0.002 to 0.02), with a reciprocal decrease in urgent visits, from 30% to 29%. Among visits in urban EDs, the proportion of visits seen only by MLPs increased from 0.7% (95%CI 0.4–1.1%) in 1995 to 5.7% (95%CI 4.0–7.4%) in 2009 (slope, 0.004; 95%CI 0.002 to 0.005), whereas the proportion of MLP-only visits among nonurban visits was less marked, increasing by about 50% from 3.0% in 1995 to 5.8% in 2009 (slope, 0.003; 95%CI –0.001 to 0.007).

## Discussion

During 1993 to 2009, there were 1.8 billion ED visits, of which 8.4% were managed at least partly by MLPs. Although there are differences in the penetrance of MLPs into US emergency care (e.g., with regard to type of mid-level, hospital, and setting), we found that ED visits seen by MLPs have increased steadily, and MLPs have become an essential part of the US emergency medicine workforce. MLPs provided care for 4.1% of ED visits in 1995 and this rose to 14.5% in 2009. Furthermore, those visits managed by MLPs only increased from 1.1% to 5.7% during the same time period.

These findings support our earlier NHAMCS analysis [10] and suggest that, with continued annual growth of ED visits, expansion of care to non-physicians is inevitable since there are insufficient physicians to fully staff US EDs, let alone mandate staffing by emergency medicine board-certified physicians only [13]. The physician shortage is likely to continue for several decades [3], particularly given the difficulty of incenting physicians to take positions in rural EDs [14]. In addition, the growing pressures to reduce costs can be addressed by hiring more MLPs, who are less expensive providers. On the positive side, workforce expansion with MLPs might improve quality and safety of care by improving such metrics as door-to-provider time and percentage of patients who leave the ED without being evaluated. Prior studies have found that ED patients are satisfied with the care provided by MLPs [15,16] and that few prefer to wait longer to be seen by a physician [15]. Nevertheless, quality concerns remain [8].

MLPs participate in the care of low acuity visits, but they also care for sicker patients (as measured by ambulance usage and admission), albeit at a lower rate than among visits seen by physicians. Despite statistical significance, these two markers of ED acuity did not materially change during the study period. Nevertheless, because the overall number of ED visits has steadily increased, even small percentages imply that large absolute numbers of higher acuity patients are being managed by MLPs alone. While this may not be ideal, it may be necessary to keep up with the growing demand for emergency care.

The role of PAs in US EDs continues to outpace that of NPs. PA involvement has grown from 2.9% in 1995 to 9.9% in 2009 while NP involvement rose from 1.1% to 4.7% during the same interval. In addition, PA-only visits continued to rise through 2009, while NP-only visits may have begun to level off (Figure 1). The shorter training period needed to gain a PA license, as compared with first obtaining an RN and then proceeding with specialized NP training, and marked expansion of PA graduates during the last decade of the twentieth century [17] probably contribute to this finding.

The study has some potential limitations. NHAMCS is subject to the limitations of survey research, with possible errors in data collection and coding. In particular, data abstractors may have recorded incorrect or incomplete data on the type of ED provider(s) seen. However, data collection errors of the NHAMCS should not vary significantly between years and likely would have limited impact on trend analysis. In addition, because NHAMCS provided only visit-level weights from 1993–2009 and added ED-level weights in 2005, we cannot yet evaluate whether temporal trends relate to more EDs hiring MLPs or increased use of MLPs in hospitals that already employed them; with additional years of data, these analyses should become possible.

In summary, MLP use continues to rise in US EDs, with greater increases seen for PAs than NPs. By 2009, approximately one in seven ED visits involved a MLP, with a substantial number seen by MLPs alone. MLPs have established themselves as a major part of the EM workforce, and although visits seen by MLPs only are generally of lower acuity, their autonomous care of more serious cases confirms that their role extends beyond minor presentations. As such, the onus will be on emergency physicians and their professional societies to provide MLPs with adequate supervision when needed and more widely available educational opportunities. The implications of these ED staffing trends for quality of care and cost-effectiveness merit further study.

## Abbreviations

ED, emergency department; MLP, mid-level provider; PA, physician assistant; NP, nurse practitioner; NHAMCS, National Hospital and Ambulatory Medical Care Survey; CI, confidence interval; IQR, interquartile range.

## Competing interests

The authors declare that they have no competing financial or non-financial interests.

## Authors’ contributions

DFMB conceived of the study, participated in its design and coordination, and drafted the manuscript. JAE participated in the design of the study, acquired the data, performed the statistical analysis, and helped to draft the manuscript. AFS participated in the design of the study and revised the manuscript. CAC conceived of the study, participated in its design and coordination, and revised the manuscript. All authors read and approved the final manuscript.

## Funding

None
